# The association between socioeconomic status and reactions to radiation exposure: a cross-sectional study after the Fukushima Daiichi nuclear power station accident

**DOI:** 10.1371/journal.pone.0205531

**Published:** 2018-10-31

**Authors:** Taro Kusama, Jun Aida, Toru Tsuboya, Kemmyo Sugiyama, Takafumi Yamamoto, Ayaka Igarashi, Ken Osaka

**Affiliations:** Department of International and Community Oral Health, Tohoku University Graduate School of Dentistry, Sendai, Miyagi, Japan; Universidad Miguel Hernandez de Elche, SPAIN

## Abstract

Risk perception and individual reactions to risk are not necessarily comparable, and socioeconomic status may affect individual reactions to risk. This study aimed to investigate the association between socioeconomic status and reactions to radiation exposure risk. This cross-sectional study, based on a self-reported online survey was conducted between 3 March and 21 March 2012, one year after the accident at Fukushima Daiichi nuclear power station. We used feelings of anxiety and risk-averse behavior concerning radiation exposure as dependent variables, and equivalent income and educational attainment as independent variables. Multiple logistic regression analysis was applied to estimate adjusted odds ratios (aORs) and 95% confidence intervals (95% CIs) with adjustment for possible confounders. Among 10 000 participants, 23.0% felt anxious and 12.0% engaged in risk-averse behavior for radiation exposure. Participants with a higher socioeconomic status tended not to feel anxious but undertook risk-averse behavior. Participants in the highest quartile income category did not report feeling anxious but showed prevalent undertaking of risk-averse behavior for radiation exposure compared to the lowest income category (for anxiety, aOR, 0.77; 95% CI, 0.64–0.93, for risk-averse behavior, aOR, 1.33; 95% CI, 1.04–1.69). University or graduate-school graduates were associated with greater risk-averse behavior compared to junior high school or high school graduates (aOR, 1.49; 95% CI, 1.29–1.73). Socioeconomic status may affect reactions to radiation exposure risk. Risk communication strategies should consider the socioeconomic status of those affected.

## Introduction

The dispersal of radioactive material and radioactive contamination following nuclear power station accidents has occasionally occurred internationally[1,2]. The spreading of contamination has resulted in health issues and, even when the contamination has been minimal, it has resulted in some people experiencing serious anxiety because communication informing affected populations of levels of risk has not been effective. Harmful rumors also affect the economy of an affected area, and may also disrupt the reconstruction of an area[3,4]. The tsunami that followed the Great East Japan Earthquake in 2011 destroyed the Fukushima Daiichi nuclear power station, and radioactive substances were spread to neighboring municipalities[5]. In most of the neighboring municipalities, the radiation level did not reach a health-threatening level, although the amount of radiation close to the power station was high[6]. However, some residents in municipalities with low radiation levels have worried about the adverse effects of radiation exposure and many have reported psychological stress due to the risks of radiation exposure[7]. Some residents have remained hesitant to purchase food items that might be contaminated with radioactive substances, even when food items have been checked for radiation prior to going on sale in markets[8]. Despite ongoing activities aiming to communicate levels of risk, anxiety and risk-averse behaviors for radiation exposure have been observed following the accident at the Fukushima Daiichi nuclear power station. These reactions suggest that effective risk communication is difficult to achieve, even when the radiation level is low.

Previous studies concerning various risk situations have reported that the reaction to certain risks differs with individual characteristics, and some individuals have difficulty determining the perception of risk[9–13]. Therefore, understanding the characteristics of those who have difficulty in risk perception is important when implementing adequate risk communication. In a health crisis, risk communication is necessary to communicate the right information, such as in situations of radioactive contamination[14]. Previous studies have shown that reactions to genetically modified food and to terrorist attacks have been associated with individual characteristics such as sex, ethnicity, educational attainment, and income[11–13,15]. However, the results of these studies have been inconsistent, which suggests that the difference in anxiety and risk-averse behaviors due to individual characteristics differs according to the type of risk. To the best of our knowledge, no study has examined the differences between anxiety and risk-averse behaviors and the socioeconomic status (SES) of residents in radiation-exposed communities. This study aimed to investigate the association between SES and anxiety and risk-averse behavior for radiation exposure one year after the Fukushima Daiichi nuclear power station accident.

## Materials and methods

### Settings and participants

We conducted a cross-sectional study using an online survey, from 3–21 March 2012, and data were obtained from responses to self-reported questionnaires. This survey was performed almost one year after the Great East Japan Earthquake that occurred on 11^th^ March 2011. We conducted the survey just one year after the disaster because people’s attention to it was re-increasing due to its one-year-period broadcastings. The study participants were those who completed the online survey; 10,000 participants living in Japan and aged between 20 and 69 years were included. The participants were randomly selected from a registry established by the Internet research company, and those who agreed to answer the questionnaire after reading the explanation participated in the survey. The online survey was closed when the number of participants reached 10,000. The same number of participants in each sex and age category was included in the survey, and almost the same number of participants was surveyed in each of the 47 prefectures in Japan (in each prefecture, n = 212 or n = 213). Using an electronic questionnaire, participants were required to answer all the questions.

### Dependent variables

We used anxiety and risk-averse behavior for radiation exposure as dependent variables. To determine anxiety concerning radiation exposure, participants were asked: “How do you feel about radiation exposure in your daily life?”, with the following choice of answers: “It is very harmful to my health”, “I don’t know much about it”, or “It is not a problem in my daily life”. We considered those who answered “It is very harmful for my health” were feeling anxious, and those who answered “I don’t know much about it” or “It is not a problem in my daily life” were not feeling anxious.

To determine risk-averse behavior concerning radiation exposure, we asked participants: “Do you take protective action against radiation exposure for yourself or your family?”, with the following choice of available answers: “Yes” or “No”. We considered those who answered “Yes” undertook some form of risk-averse behavior and those who answered “No” did not engage in any risk-averse behavior.

### Independent variables

We used equivalent income and educational attainment as socioeconomic independent variables. We questioned participants concerning their annual household income and the number of household members, and with this information we calculated the equivalent income. We created four categories for equivalent income, as follows: <2,000,000 Japanese Yen (JPY), from 2 000 000 JPY to <4 000 000 JPY, from 4 000 000 JPY to <6 000 000 JPY, and ≥6 000 000 JPY. One hundred JPY was equal to 1 US dollar (USD).

We created four categories for educational attainment: junior high school or high school graduates, vocational school or junior college graduates, university or graduate school graduates, and others. If school students were surveyed, they were required to identify the school that they attended.

### Covariates

We used possible confounders as covariates. Confounders were selected from related studies investigating the association between reaction to uncertain risk and individual characteristics[6,12,13,16–18] such as sex, age, and residential area. We categorized age into five groups as follows: 20–29 years, 30–39 years, 40–49 years, 50–59 years, and 60–69 years. Residential area was categorized into two groups, that is, one group comprised participants either living in the Fukushima prefecture and the other group comprised participants who did not live in the Fukushima prefecture.

### Statistical analysis

We calculated adjusted odds ratios (aORs) and 95% confidence intervals (95% CIs) using multiple logistic regression analysis. We made three models in the analysis. Model 1 comprised univariate models, each including independent variables only (including equivalent income or educational attainment only). Model 2 included each independent variable and was adjusted for all covariates (all covariates were added to Model 1). Model 3 included both equivalent income and educational attainment variables and was adjusted for all covariates. We used Stata MP version 14.1 (Stata Corp., College Station, TX, USA) for statistical analysis.

### Ethical issue

In this study, the process of informed consent was as follows: In this online survey, we explained the survey to the participants via the Internet, and those who read the online explanation and agreed to participate answered the online questionnaire. This study was approved by the Ethics Committee of Tohoku University Graduate School of Dentistry (ID:2015-3-21).

## Results

From a target population of 10 000 people, all participants responded to the online survey. Table 1 shows the descriptive characteristics of the participants feeling anxious and engaging in risk-averse behavior. The mean age and standard division (SD) of the participants was 44.4 years (SD = 13.5). Of these, 5 000 (50.0%) participants were male. Among 10 000 participants, 2 302 (23.0%) reported feelings of anxiety concerning radiation exposure, and 1 203 (12.0%) participants undertook risk-averse behavior. The ratios of those who reported feeling anxious regarding radiation exposure were higher among lower income participants. The proportion of those who had engaged in risk-averse behavior was higher among those with a higher educational attainment.

**Table 1:** Socioeconomic characteristics of participants and reaction to radiation exposure (N = 10,000).

Characteristics	Anxiety for radiation exposure				Risk-averse behavior for radiation exposure			
	Yes		No.		Yes		No	
	N	%	N	%	N	%	N	%
Equivalent income								
<2,000,000 JPY	631	25.9	1,808	74.1	268	11.0	2,171	89.0
2,000,000≤ <4,000,000 JPY	1,062	22.3	3,696	77.7	558	11.7	4,200	88.3
4,000,000≤ <6,000,000 JPY	425	22.3	1,477	77.7	261	13.7	1,641	86.3
≥6,000,000 JPY	184	20.4	717	79.6	116	12.9	785	87.1
Educational attainment								
Juniour high school or High school graduate	790	23.5	2,577	76.5	349	10.4	3,018	89.6
Vocational school or Junior college graduate	557	23.4	1,820	76.6	306	12.9	2,071	87.1
University or Graduate-school graduate	951	22.4	3,293	77.6	548	12.9	3,696	87.1
Others	8	66.7	4	33.3	0	0.0	12	100.0

Table 2 shows the association between feeling anxious in respect of radiation exposure and SES using multiple logistic regression analysis. In the univariate and multivariate adjusted models, equivalent income was significantly associated with feelings of anxiety concerning radiation exposure while educational attainment was not (models 1 and 2), and participants with a higher income tended to report fewer feelings of anxiety (*p* for trend = 0.006). When including both SES measures simultaneously, a significant association of income was still observed (model 3).

**Table 2:** The association between anxiety for radiation exposure and socioeconomic status (N = 10,000).

	Model 1*		Model 2**		Model 3***	
	OR (95% CI)	*p* value	aOR (95% CI)	*p* value	aOR (95% CI)	*p* value
Equivalent income						
<2,000,000 JPY	Ref.		Ref.		Ref.	
2,000,000≤ <4,000,000 JPY	0.82 (0.74–0.92)	0.001	0.83 (0.74–0.93)	0.002	0.83 (0.74–0.93)	0.002
4,000,000≤ <6,000,000 JPY	0.82 (0.72–0.95)	0.007	0.86 (0.74–0.99)	0.042	0.86 (0.74–0.995)	0.043
≥6,000,000 JPY	0.74 (0.61–0.89)	0.001	0.77 (0.64–0.93)	0.007	0.77 (0.64–0.93)	0.008
Educational attainment						
Junior high school or High school graduate	Ref.		Ref.		Ref.	
Vocational school or junior college graduate	1.00 (0.88–1.13)	0.979	0.99 (0.87–1.12)	0.872	1.00 (0.88–1.13)	1.000
University or graduate-school graduate	0.94 (0.85–1.05)	0.277	0.98 (0.88–1.10)	0.769	1.01 (0.91–1.14)	0.808

*Model 1: Univariate model

**Model 2: Adjustment for age, sex, and residential area (equivalent income and educational attainment variables were separately included)

***Model 3: Included both equivalent income and educational attainment variables and all covariates

Table 3 also shows the association between risk-averse behavior in relation to radiation exposure and SES using multiple logistic regression analysis. In the univariate and multivariate adjusted models, equivalent income and educational attainment were significantly associated with risk-averse behavior in relation to radiation exposure while equivalent income was not (model 1 and 2). Participants with higher educational attainment and higher income tended to engage in risk-averse behavior (both *p* for trend < 0.001). When we included both SES measures simultaneously, a significant association between equivalent income and educational attainment was still observed (model 3).

**Table 3:** The association between risk-averse behavior for radiation exposure and socioeconomic status (N = 10,000).

	Model 1*		Model 2**		Model 3***	
	OR (95% CI)	*p* value	aOR (95% CI)	*p* value	aOR (95% CI)	*p* value
Equivalent income						
<2,000,000 JPY	Ref.		Ref.		Ref.	
2,000,000≤ <4,000,000 JPY	1.08 (0.92–1.26)	0.352	1.10 (0.94–1.29)	0.242	1.06 (0.91–1.25)	0.445
4,000,000≤ <6,000,000 JPY	1.29 (1.07–1.54)	0.006	1.41 (1.18–1.71)	<0.001	1.31 (1.08–1.59)	0.006
≥6,000,000 JPY	1.20 (0.95–1.51)	0.13	1.33 (1.04–1.69)	0.021	1.18 (0.92–1.51)	0.186
Educational attainment						
Junior high school or High school graduate	Ref.		Ref.		Ref.	
Vocational school or junior college graduate	1.28 (1.09–1.50)	0.003	1.17 (0.99–1.39)	0.065	1.16 (0.97–1.37)	0.096
University or graduate-school graduate	1.28 (1.11–1.48)	0.001	1.49 (1.29–1.73)	<0.001	1.43 (1.23–1.67)	<0.001

*Model 1: Univariate model

**Model 2: Adjustment for age, sex, and residential area (equivalent income and educational attainment variables were separately included)

***Model 3: Included both equivalent income and educational attainment variables and all covariates

## Discussion

This study revealed the association between SES and radiation exposure-related reactions, namely, that lower income participants tended to feel anxious regarding radiation exposure, and those who had a higher equivalent income or educational attainment tended to engage in risk-averse behavior in relation to radiation exposure. The results of this study suggest that risk communication activities provided following the Fukushima Daiichi nuclear power station accident were not sufficiently targeted, and feeling anxious and engaging in risk-averse behavior were evident in differing proportions between social groups.

Those who had lower incomes tended to feel anxious about radiation exposure. Among the general population, previous studies have shown that those who have a low SES or a low income tend to feel psychological distress and are depressed[19,20]. The proportion of those who have trust in governments is low among the low-income population, and it is possible that people on low incomes feel more anxious in disaster situations[21]. For risks that may arise regarding food, those on low incomes perceive risks as greater than those on higher incomes[22]. Risk perception is affected through individual characteristics such as the “White Male Effect”[23]. Further studies have revealed other social backgrounds affect risk perception[24,25]. A previous study investigated evacuees from the neighboring area of Fukushima Daiichi nuclear power station and showed that those who had lower educational attainment tended to have feelings of anxiety regarding radiation exposure[7]. Here, we included two SES variables; income and educational attainment, and only income was significantly associated with feeling anxious. It is possible that the mechanism involved is that people on a low income are usually more disadvantaged, and individuals with a low SES, especially those with a low income have, therefore, an increased perception of risk leading to a higher prevalence of anxiety among the low-income population. Consequently, in disaster situations which involve the possibility of adverse effects on health, income levels are likely to affect anxiety levels.

In this study, those who had higher income or educational attainment tended to engage in risk-averse behavior in relation to radiation exposure. The association between SES and risk-averse behavior has not been consistent among previous studies. Several studies have shown that educational attainment and household income were not associated with behavioral change to avoid the risks from terrorism in daily life[13,16]. For genetically modified food, previous studies have shown that those with higher educational attainment tended to accept genetically modified food[15,26]. Generally, SES influences changes in behavior and several models of change behavior have been suggested. The process whereby people alter their behavior towards more health-protective behavior has been outlined in the Knowledge, Attitude, and Behavior Model (KAB model)[27,28]. Previous studies have reported that those with a higher educational attainment were more likely to participate in health-protective behavior[29–31]. Here, those who had higher educational attainment had more knowledge of radiation exposure, and they may have changed their behavior to avoid risks of radiation exposure. Participants with a higher equivalent income also tended to engage in risk-averse behavior. One study reported that those who had higher incomes were less likely to accept food produced near the Fukushima Daiichi nuclear power station[3]. Those who had higher incomes could afford to pay more to avoid risks of radiation. In contrast, no associations between SES and risk-averse behavior were identified in analyzing the risk of terrorism, which appears to be more difficult to predict, and associations between SES and risk-averse behavior in terms of food contamination and risk of radiation were more likely to occur because these risks are easier to manage.

As an implication of this study, we need to take the SES of individuals SES into consideration when communicating on levels of risk. The range and level of radiation occurring after the Fukushima Daiichi nuclear power station accident was below the level of having an adverse effect on health. No one needed to feel anxious or engage in risk-averse behavior in reaction to radiation exposure. According to the results of this study, those with low incomes and in socially vulnerable positions were more likely experience feelings of anxiety in critical situations, and those with higher incomes or educational attainment were more likely to engage in risk-averse behavior. It has been reported that socially vulnerable people were more likely to be victims in situations of crisis such as disasters or epidemics[32–34]. Previous studies suggested that risk communication considering the SES of individuals is necessary[21,35]. In future, governments and municipalities need to conduct appropriate and effective risk communication for low-SES populations in critical situations following disasters because socially vulnerable people appear are more likely to be affected by a disaster. Additionally, risk-averse behavior such as avoiding purchasing food produced in the neighboring area where the accident occurred may lead to harmful rumors and may also delay the reconstruction of the affected area. One previous study suggested that effective risk communication could reduce the anxiety of those affected by a disaster[36].

One limitation of this study is that it was based on a self-reported questionnaire, and we were unable to evaluate whether participants engaged in risk-averse behavior or not. This study was also based on an online survey, and all participants had access and were able to use the internet. Therefore, they could easily have obtained correct and incorrect information from an internet web site, and this may have resulted in an imbalanced directional bias. The strength of this study is that we were able to obtain a sample from all prefectures within a short timeframe. The actual influence of the Fukushima Daiichi nuclear power station accident was limited to a narrow area around the nuclear power station, but the psychosocial influence ranged more widely across Japan. Most previous studies that had investigated the psychosocial influence of the Fukushima Daiichi nuclear power station accident included only the population that lived in the Fukushima prefecture and/or the main urban area, and few studies have included people living across all regions of Japan. Furthermore, few studies have investigated the association between SES and reactions to radiation exposure simultaneously involving factors such as feeling anxious and engaging in risk-averse behavior[37].

### Conclusions

This study revealed that lower-income participants tended to feel anxious concerning radiation exposure, and those who had a higher income or educational attainment tended to engage risk-averse behavior for radiation exposure. Although the level of radiation exposure after the Fukushima Daiichi nuclear power station accident was below the level considered to have an adverse effect on health, feeling anxious and risk-averse behavior were observed, and these reactions differed according to SES. To reduce unnecessary feelings of anxiety and avoid disruption to the reconstruction of an affected area because of harmful rumors in these types of critical situations, governments and municipalities should conduct appropriate risk communication strategies that take into consideration the SES of the affected population.

## Supporting information

S1 File (DOCX)
