# Supplementary material for: The association between socioeconomic status and reactions to radiation exposure: a cross-sectional study after the Fukushima Daiichi nuclear power station accident
Source: PLoS One. 2018 Oct 31;13(10):e0205531. doi: 10.1371/journal.pone.0205531 (PMC6209195; doi:10.1371/journal.pone.0205531)
Supplement: S1 File — (DOCX) [file pone.0205531.s001.docx]

**Supporting Information**

Our questionnaire aimed to obtain various information. Each question measured different contents. Therefore, validation of questions referring to a single concept was not adequate. For socioeconomic questions, widely used questions from other surveys were used. For other questions, because they were unique and newly constructed, researchers discussed about the validity before they were finalized.

**The original survey questions in Japanese**

For anxious for radiation exposure

Question:

現在あなたが生活する上で生じている放射性物質の被ばく（内部被ばく・外部被ばく問わず）について、どのように感じていますか?

Answer:

1. あまりわからない

2. 日常生活を送る上では問題ないと思う

3. とても健康に悪いと思う

For risk-averse behavior for radiation exposure

Question:

放射線被ばくを防ぐために、自分や家族のために行っていることはありますか？

Answer:

1. ある 2. ない

For house hold income

Question:

世帯全体の合計収入額（税引き前。年金を含みます。）は、過去１年間で、次のうちどれにあてはまりますか？

Answer:

1. 50万未満

2. 50～100万円未満

3. 100～200万円未満

4. 200～300万円未満

5. 300～400万円未満

6. 400～500万円未満

7. 500～600万円未満

8. 600～700万円未満

9. 700～800万円未満

10. 800～900万円未満

11. 900～1000万円未満

12. 1000万円以上

For the number of house hold member

Question:

同居している家族は何人ですか?（自分を含めた人数）／人

Answer:

直接数値を入力

For the educational attainment

Question:

あなたの最終学歴を教えてください？

Answer:

1. 中学校卒業

2. 高校卒業

3. 専門学校卒業（在学含む）

4. 短期大学／高等専門学校　卒業（在学含む）

5. 大学卒業（在学含む）

6. 大学院卒業（在学含む）

7. その他

**The original survey questions translated in English**

For anxious for radiation exposure

Question:

How do you feel about radiation exposure in your daily life?

Answer:

1. I don’t know much about it.

2. It is not a problem in my daily life.

3. It is very harmful to my health.

For risk-averse behavior for radiation exposure

Question:

Do you take protective action against radiation exposure for yourself or your family?

Answer:

1. Yes. 2. No.

For house hold income

Question:

Which following answers is most suited for your last year-household income (pre-tax and including pension)? (JPY: Japanese yen)

Answer:

1. under 50 JPY

2. 500 000 to <1 000 000 JPY

3. 1 000 000 to <2 000 000 JPY

4. 2 000 000 to <3 000 000 JPY

5. 3 000 000 to <4 000 000 JPY

6. 4 000 000 to <5 000 000 JPY

7. 5 000 000 to <6 000 000 JPY

8. 6 000 000 to <7 000 000 JPY

9. 7 000 000 to <8 000 000 JPY

10. 8 000 000 to <9 000 000 JPY

11. 9 000 000 to <10 000 000 JPY

12. 10 000 000 and over JPY

For the number of house hold member

Question:

Please, answer the number of person live with you (including yourself).

Answer:

Participants had to input the number directory.

For the educational attainment

Question:

Please, answer your educational attainment.

Answer:

1. Junior high school graduate

2. High school graduate

3. Vocational school graduate (including those who are in school)

4. Junior college technical college graduate (including those who are in school)

5. University graduate (including those who are in school)

6. Graduate school graduate (including those who are in school)

7. The other
